# Why do people vary in disgust?

**DOI:** 10.1098/rstb.2017.0204

**Published:** 2018-06-04

**Authors:** Joshua M. Tybur, Çağla Çınar, Annika K. Karinen, Paola Perone

**Affiliations:** 1 Department of Experimental and Applied Psychology, 1081 HV Amsterdam, The Netherlands; 2 Institute of Brain and Behaviour, Amsterdam, The Netherlands

**Keywords:** pathogen avoidance, disgust, behavioural immune system, personality, behavioural genetics

## Abstract

People vary in the degree to which they experience disgust toward—and, consequently, avoid—cues to pathogens. Prodigious work has measured this variation and observed that it relates to, among other things, personality, psychopathological tendencies, and moral and political sentiments. Less work has sought to generate hypotheses aimed at explaining why this variation exists in the first place, and even less work has evaluated how well data support these hypotheses. In this paper, we present and review the evidence supporting three such proposals. First, researchers have suggested that variability reflects a general tendency to experience anxiety or emotional distress. Second, researchers have suggested that variability arises from parental modelling, with offspring calibrating their pathogen avoidance based on their parents' reactions to pathogen cues. Third, researchers have suggested that individuals calibrate their disgust sensitivity to the parasite stress of the ecology in which they develop. We conclude that none of these hypotheses is supported by existing data, and we propose directions for future research aimed at better understanding this variation.

This article is part of the Theo Murphy meeting issue ‘Evolution of pathogen and parasite avoidance behaviours’.

## 1. Introduction

In the traditional layout of university campuses and curricula, parasitology and psychology have been separated by a gulf no smaller than that which separates mathematics from English literature and chemistry from sociology. Nevertheless, scientists have discovered natural bridges that connect the study of infectious microbes to the study of human behaviour—bridges that are undergirded by evolutionary theory. Research into the evolutionary arms races between parasites and hosts has led to the discovery of some adaptations that facilitate infections and others that neutralize them. At the same time, an increased understanding that the human mind is composed of myriad mechanisms specialized for navigating the types of threats and affordances faced by human ancestors has spurred the development of flourishing evolutionary psychology research programmes. And, hence the bridge: to a parasitologist, humans should have anti-pathogen adaptations, just as other species do; to an evolutionary psychologist, some of our modular psychological mechanisms should be specialized to neutralizing pathogens, just as other mechanisms are specialized for neutralizing aggressive conspecifics or identifying, acquiring and retaining a mate. These insights have served as a kind of treasure map for psychologists—they have suggested that anti-pathogen psychological adaptations lie waiting to be discovered, and they have hinted at how to find such adaptations.

Initial forays into understanding human anti-pathogen adaptations have examined people's responses to the types of substances and organisms that reliably housed or transmitted pathogens over our ancestral history (e.g. faeces, vomit, spoiled food, blood, saliva and arthropod vectors such as ticks and fleas). As it happens, we reliably experience disgust—which some prominent models of emotion have categorized as a universal and ‘basic’ emotion (e.g. [1])—toward such substances [2]. After identifying disgust as a candidate anti-pathogen adaptation, researchers have further investigated whether it demonstrates good (i.e. specialized) fit for this function [3–5]. It does. The facial movements that accompany disgust limit the degree to which the surface of the eye is exposed to those pathogens that infect via sprays of liquid from an already infected individual ([6]; notably, the reduced surface area of the eyes has alternatively been interpreted as functioning to increase visual acuity so that potentially pathogenic substances can be better examined [7]). If sensory cues to pathogens are detected in the mouth (e.g. via taste perceived as disgusting), then the tongue expels them; if pathogen cues are detected via olfaction, then the lips clench to prevent oral incorporation [8]. And, perhaps most importantly, disgust motivates the avoidance of physical contact with the disgust elicitor—exactly the type of contact that would transmit pathogens [9]. Based on these types of observations, researchers have argued that disgust is a central component of the human behavioural immune system [10,11].

**Figure 1.**
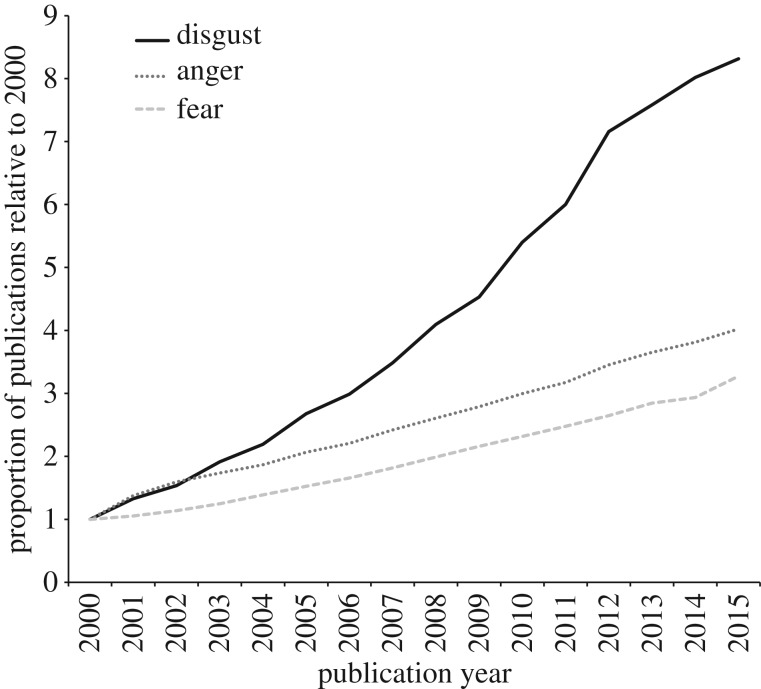
Increase in public work on disgust, anger and fear indexed by Thomson Reuter Web of Science from the year 2000 to 2015. Each emotion record is proportional to the number of search hits for the year 2000, and each represents a 3-year average. Year 2000 hits for disgust were 59, for anger 471 and for fear 2571.

With the recognition that disgust is an anti-pathogen adaptation, evolutionary psychologists have increasingly aimed to measure the emotion to test pathogen-avoidance hypotheses. At the same time (and, at times, running parallel to work guided by evolutionary theory) social and clinical psychologists have found that feelings of disgust seem to underlie many moral judgements, prejudices and psychopathologies—findings that make sense given the importance of neutralizing pathogens in so many life domains (e.g. social interaction, food choice, sexual behaviour [12–14]). Together, such investigations have led to an explosion of disgust research over the first 15 years of the twenty-first century. Indeed, the acceleration of disgust research has far outpaced increases in research on other emotions, such as anger and fear (figure 1). Some of this work has presented theoretical accounts of the emotion [5,10,15]. Other work has measured facial movements (e.g. of the levator labii, a muscle activated when people experience disgust) to test whether people experience more disgust in some contexts than in others, such as when calorically deprived versus sated [16]. But the lion's share of disgust research has examined traits known as disgust sensitivity^1^ (or disgust propensity; see [17] for a discussion of terminology) and contamination sensitivity [19]. For example, studies have measured disgust sensitivity to test hypotheses that pathogen-avoidance adaptations influence colour discrimination [20], person perception [21] and orientations towards gregariousness versus introversion [22]. To give an indication of the widespread use of these instruments, the papers developing the Disgust Scale [23], the Disgust Scale—Revised [24], the Three Domain Disgust Scale [18] and the Perceived Vulnerability to Disease scale [25] have been cited 1287, 447, 502 and 202 times at the time of this writing, respectively (see table 1 for a description of these scales).

**Table 1.** Instruments widely used in the disgust sensitivity literature.

instrument	subscale	no. items	highest loading item	interpreted as measuring pathogen avoidance
Disgust Scale–Revised	core	12	It would bother me to see a rat run across my path in a park	yes
	contamination	5	A friend offers you a piece of chocolate shaped like a dog-doo	yes
	animal reminder	8	It would bother me tremendously to touch a dead body	no
Three Domain Disgust Scale	pathogen	7	standing close to a person who has body odour	yes
	sexual	7	bringing someone you just met back to your room to have sex	no (though see [26,27])
	moral	7	forging someone's signature on a legal document/intentionally lying during a business transaction	no (though see [26,27])
Perceived Vulnerability to Disease scale	germ aversion	8	I prefer to wash my hands pretty soon after shaking someone's hand	yes
	perceived infectability	7	In general, I am very susceptible to colds, flu and other infectious diseases	no

Relative to the volume of research measuring disgust sensitivity, little work has aimed to better understand why this variation exists in the first place. Greater disgust sensitivity is not cost-free, after all; it is associated with greater avoidance of activities, people and resources (e.g. foods) that, while potentially pathogenic, are also potentially beneficial [24,28,29]. So why do some people appear to be more motivated to avoid pathogens than others? We have identified three categories of proposed answers to this question: (i) disgust sensitivity is an epiphenomenon of broader emotionality or neuroticism; (ii) disgust sensitivity is shaped early in life by parental modelling; and (iii) disgust sensitivity is shaped by earlier exposure to pathogens. In the following sections, we summarize these proposals and evaluate each based on data that have accumulated in the literature. We then provide proposals that can further evaluate these and alternative hypotheses.

## 2. Is disgust sensitivity an epiphenomenon of broader emotionality?

Galton [30] suggested that the general dimensions of human personality (or ‘character’) can be uncovered by analysing the types of words that people use to describe each other. That is, the degree to which people's ‘cooperativeness’ relates to their ‘courteousness’, ‘empathy’ and ‘cruelty’ can inform the presence of an underlying personality factor. This lexical hypothesis has formed the foundation of personality psychology [31], and factor analyses on adjectives across multiple cultures and languages have revealed the presence of five [31] or six [32] factors. Both five- and six-factor models (and, further, earlier models based less on lexical traditions [33]) include a personality dimension labelled as neuroticism or emotionality, with adjectives that define this factor including ‘anxious’, ‘fragile’, ‘emotional’ and ‘fearful’ [32]. Since the publication of the Disgust Scale [23], researchers have proposed that disgust sensitivity should relate to neuroticism/emotionality. Indeed, one account stated ‘it is probable that disgust sensitivity is, in fact, a component of the neuroticism trait’ ([10], p. 392). If correct, this proposal carries at least two important implications for the disgust literature, and, further, for how we understand variability in human pathogen avoidance. First, many of the conclusions reached based on disgust sensitivity research might need to be reinterpreted as reflecting broader emotionality rather than pathogen avoidance. And second—and of particular importance to our understanding of the roots of variation in disgust sensitivity—the same factors that shape variability in neuroticism should also shape variability in disgust sensitivity (e.g. [34]). But how well do the data support this proposal?

### (a) The relationship between disgust sensitivity and neuroticism/emotionality

In developing the original Disgust Scale, Haidt *et al.* [23] predicted that disgust sensitivity (as measured by a preliminary form of the Disgust Scale) would covary with neuroticism. And, indeed, they observed a modestly sized relationship (*r* = 0.23) between the two variables (with neuroticism measured using the Eysenck Personality Questionnaire) in a sample of 124 college students. Some subsequent studies reported stronger relationships, with the Disgust Scale correlating with NEO-PI-R neuroticism at *r* = 0.45 in a sample of 132 college students [35], and NEO-FFI neuroticism correlating with the Disgust Scale–Revised at *r* = 0.46 in a sample of 247 college students [24]. Relationships of this magnitude have not been observed in larger samples with other disgust instruments, though. In developing the Three Domain Disgust Scale, Tybur and colleagues [18] found that disgust sensitivity was only weakly related to BFI neuroticism, *r* = 0.15, in a sample of 300 college students. This weaker relationship was later corroborated in larger studies finding that disgust sensitivity relates only weakly to NEO-PI-3 neuroticism, *r* = 0.10 [36], BFI neuroticism, *r* = 0.10 [26], HEXACO emotionality, *r* = 0.23, and 5DPT neuroticism, *r* = 0.13 [37]. Further, in developing the Perceived Vulnerability to Disease scale, Duncan and colleagues [25] found that the germ aversion subscale relates similarly weakly to BFI neuroticism (*r* = 0.17) in a sample of 661 college students; a similar effect size (*r* = 0.12) was reported between germ aversion and EPQ neuroticism in a sample of 878 Spanish students [38]. Further, other methods (e.g. self-reports of disgust toward food being spat on; eye-blind startle response toward disgust-eliciting stimuli) suggest that variability in disgust toward pathogen cues is unrelated to neuroticism [39,40].

Why have some studies found stronger relationships between disgust sensitivity and neuroticism than others? The answer might lie in subtle differences in how disgust sensitivity instruments are constructed. The Disgust Scale (and Disgust Scale–Revised), which was administered in the studies reporting the strongest relationships with neuroticism, contains multiple items (seven out of 25 in the Disgust Scale–Revised) that ask participants to indicate how ‘bothered’ or ‘upset’ they would be in the situations described in the items (e.g. ‘It would bother me to see a rat run across my path in a park’). Clark & Watson [41], in their seminal review of scale development practices, warn that such phrasing ‘virtually guarantees that an item will have a substantial neuroticism component; the inclusion of several such affect-laden items, in turn, ensures that the resulting scale—regardless of its intended construct—will be primarily a marker of neuroticism’ [41, p. 312]. Relationships between neuroticism/emotionality and instruments such as the Three Domain Disgust Scale, which does not include such phrasing, appear weaker.

In sum, while the relationship between disgust sensitivity and neuroticism/emotionality appears to be non-zero, it is modest enough to lead to the following conclusions: (1) disgust sensitivity is not a component of neuroticism/emotionality; (2) the validity of inferences based on the use of disgust sensitivity instruments (perhaps with the exception of the Disgust Scale and Disgust Scale–Revised) is unlikely to be compromised by confounds with neuroticism/emotionality; and (3) neuroticism/emotionality and disgust sensitivity likely have different genetic and environmental roots. What are these factors that shape disgust sensitivity, though? That is, why do some people end up responding to pathogen cues with intense disgust, whereas others end up responding with little disgust? In the next section, we discuss existing proposals in the literature, and we describe recent evidence that can evaluate these proposals.

## 3. Is disgust sensitivity shaped by parental modelling?

Given that disgust sensitivity is associated with some negative mental health outcomes (e.g. obsessive–compulsive traits), clinicians and applied researchers have sought to better understand the developmental roots of high disgust sensitivity [42]. At the same time, emotion researchers have sought to understand how and when in their developmental trajectories people begin experiencing disgust [43]. Researchers from both perspectives have concluded that disgust sensitivity develops largely (and, perhaps, entirely) based on modelling the context and intensity of parents' disgust reactions [42,44–46]. Haidt *et al.* [23, p. 711] describe this conclusion straightforwardly: ‘disgust sensitivity appears to be the product of socialization’.

Two lines of evidence form the foundation of the socialization account of disgust sensitivity. First, children do not appear to experience disgust until around age 5 [43], after years of observing parents expressing disgust. Second, parents and offspring score similarly on measures of disgust (or contamination) sensitivity [46–48], as do sibling pairs [49]. Upon closer examination, though, neither observation provides strong support for the socialization hypothesis. Regarding the first line of evidence: in contrast with many lay intuitions (and assumptions of many scientists), the absence of a trait early in life does not provide evidence that the trait emerges from socialization [50]. As noted by Tooby & Cosmides [51], traits such as teeth, beards and breasts are absent at birth but reliably develop independently of the environmental inputs that would be classified as ‘socialization’. Similarly, traits that could be classified as ‘psychological’ (e.g. the capacity for language acquisition, object permanence, sexual arousal) are absent at birth but reliably develop independently of socialization. Experiences of disgust—and variation in the typical intensity of these experiences—might also emerge independently of social input. Regarding the second line of evidence: within-family similarities can reflect environmental transmission from parents to offspring (e.g. socialization) or genetic transmission from parents to offspring. Hence, any similarity between parents and offspring in contamination sensitivity [47] or expressed disgust toward pathogen cues [46] could reflect shared genes between parents and offspring rather than learning transmitted from parents to offspring.

How can researchers determine the degree to which within-family similarities in a trait reflect the influence of genetic factors versus environmental ones, which include parental socialization? The classic twin design provides an elegant method of estimating the magnitude of these influences [50]. Correlations between monozygotic (identical) twins emerge because twins share both genes and environments (e.g. parental socialization). These same genetic and environmental factors underlie correlations between dizygotic (fraternal) twins. However, any shared genetic effects should be half as strong for dizygotic twins as they are for identical twins. Hence, correlations within monozygotic twin pairs can be compared with correlations within dizygotic twin pairs to estimate the degree to which trait variance is due to shared environmental influences, such as parental modelling. Two studies have taken this approach to understanding disgust sensitivity.

The first study was conducted on a sample of 38 monozygotic twin pairs and 34 dizygotic twin pairs [49]. It tested whether correlations within monozygotic pairs differed from correlations within dizygotic pairs on five items asking participants how much they would like to eat a food that had come into contact with other objects (e.g. a cookie that had fallen on the ground). For each of the five items, the correlations for monozygotic twins were not significantly different from the correlations for the dizygotic twins. This result was interpreted as suggesting ‘that heredity has minimal effects, in comparison to family environment’ [49, p. 133]. However, the study was woefully underpowered to detect genetic effects. To understand why, consider a larger twin study of personality (and, specifically, for this example, neuroticism) testing 123 monozygotic twin pairs and 127 dizygotic twin pairs [52]. Using more advanced latent variable estimates of heritability, the authors found that genes accounted for 41% of the variance in neuroticism (and, notably, that shared environment had no influence on neuroticism). Here, monozygotic twins' neuroticism correlated *r* = 0.41, whereas dizygotic twins’ neuroticism correlated *r* = 0.18. If the population correlations investigated by Rozin & Millman [49] were similar in magnitude, then Rozin & Millman's sample size and analytic approach yielded under 18% power^2^ to detect genetic effects. Findings such as those reporting that disgust sensitivity covaries with 6-n-propylthiouracil (PROP) taste sensitivity [54], which is mostly influenced by genes and minimally by shared environment [55], further suggest that these early findings be viewed with scepticism. Ultimately, while a commendable step in the direction of disentangling the influence of genes and environment in the development of disgust sensitivity, the study was unable to test its intended hypothesis.

A study conducted about 30 years later aimed to enrol a larger sample to test the relative contributions of genetic and environmental factors to disgust sensitivity [53]. In a sample of female Finnish twins, the correlation between 131 monozygotic twin pairs' pathogen disgust sensitivity was *r* = 0.49, whereas the correlation between 100 dizygotic twin pairs’ pathogen disgust sensitivity was *r* = 0.23. Based on latent variable modelling of genetic, shared and non-shared environmental influences, 50% of the variance in disgust sensitivity was due to genes, 50% was due to environmental factors that twins did not share and none of the variance was due to environmental factors that twins shared. These results are consistent with earlier findings that disgust sensitivity is similar within families [46–49], but they suggest that such findings should not be interpreted as evidence that disgust sensitivity emerges via socialization processes (cf. [23,42,44]). Of course, they should also not be interpreted as evidence that social learning is irrelevant to disgust; indeed, social learning surely leads to some objects being interpreted as infectious (e.g. individuals who are tagged by others as having some infectious disease, poor hygiene or as having some infectious disease or poor hygiene) and some foods as having sufficient nutritional resources to eat and, hence, perceived as having positive contact value relative to pathogen costs [4,5]. These same learning processes need not shape the intensity of disgust responses to such objects, though. And, of course, the fact that half of the variance in disgust sensitivity in the sample of Finnish twins was accounted for by unshared environmental factors suggests that environmental factors—even if they are not social learning—strongly shape disgust sensitivity. But what are these factors? We turn to another popular hypothesis—that disgust sensitivity is calibrated to infectious disease, either experienced personally or present in the broader ecology—in the next section.

## 4. Is disgust sensitivity shaped by infectious disease?

One line of thinking in the behavioural immune system literature suggests that disgust sensitivity should be higher for those who are relatively more vulnerable to infectious disease [56]. This proposal resonates with facultative calibration hypotheses (e.g. [57–59]), which suggest that behavioural variation partially emerges from adaptations that calibrate behaviour to an individual's condition and ecology. For example, researchers have suggested that individuals high in attractiveness or physical formidability tend to be more extraverted because the benefits of behaviours associated with extraversion are higher for such individuals and the costs are lower [57]. Considerations of the costs and benefits of high versus low disgust sensitivity have been interpreted as implying that (i) individuals in nations with greater infectious disease burden should have greater disgust sensitivity and (ii) individuals who otherwise pay higher costs for pathogen contact (e.g. due to less ability to resist pathogens) should have greater disgust sensitivity. Accumulating research allows us to evaluate these predictions.

### (a) Variability in disgust sensitivity as a function of national parasite stress

The prodigious parasite stress literature (e.g. [12,60]) has shown that many psychological traits—including higher religiosity, higher collectivism and lower openness to experience and extraversion, each of which putatively mitigates the costs of pathogens by limiting contact with individuals who are more likely to transmit infection and encouraging contact with individuals who are more likely to provide caretaking in the event of infection—are higher in nations with greater infectious disease burdens (cf. [61]). These results mirror individual-level findings that disgust sensitivity relates to, among other things, openness to experience [36], religiosity [62] and collectivism [63]. This concordance—along with the idea that people would benefit more from experiencing disgust toward pathogen cues in areas with more infectious disease—has led researchers to predict that people in nations with higher infectious disease burdens should also be more disgust sensitive. As put straightforwardly by Fincher & Thornhill [12, p. 78], ‘the relationship between infectious disease and religion will be mediated by … disgust and contamination sensitivity’.

One study tested whether individuals in a relatively high parasite stress nation (Ghana—based on a sample of 103 undergraduates) score higher on a disgust sensitivity instrument than did individuals in a relatively low parasite stress nation (USA—based on a sample of 96 undergraduates) [64]. Results suggested that disgust sensitivity is indeed higher in the nation with more infectious disease. Of course, this study was limited in that it sampled from only two nations. A recent study that measured both disgust sensitivity and traditionalism in over 11 000 individuals across 30 nations was able to provide a better test of the relationship between national infectious disease and disgust sensitivity [65]. Replicating previous cross-cultural work, individuals from nations with higher infectious disease burdens scored higher on a measure of traditionalism. And, also replicating previous work, greater disgust sensitivity was associated with greater traditionalism within nations. However, participants in nations with higher infectious disease rates were no more sensitive to disgust than were participants in nations with lower infectious disease rates. These results resonate with those reported by Curtis *et al.* [10], who found that disgust ratings of pictures (collected by Curtis *et al.* [66]) did not vary across nine world regions—including Europe, the Far East, North America and the Indian Subcontinent—in a sample of 30 839 individuals.

At first blush, failures to observe greater disgust sensitivity in nations with more infectious disease might appear to strike a blow against the hypothesis that disgust functions to neutralize pathogens and disgust sensitivity reflects motivations to avoid pathogens. Deeper considerations of both the costs and benefits of such motivations render such findings more compatible with theory. Contrast an individual in a less pathogen-rich ecology—someone who risks contact with pathogens once per day, on average—with an individual from a more pathogen-rich ecology—someone who risks contact with pathogens 50 times per day, on average. Further assume that a relatively high level of disgust sensitivity is associated with a 1% chance of contact when pathogens are detected, and a relatively low level of disgust sensitivity is associated with a 5% chance of contact when those same pathogens are detected. In the less pathogen-rich ecology, the more disgust-sensitive individual has a 7% weekly chance of contact with pathogens, and the less disgust-sensitive individual has a 30% weekly chance of contact. Here, the benefits of higher disgust sensitivity are clear. In the more pathogen-rich ecology, the more disgust-sensitive individual has a 97% weekly chance of pathogen contact, and the less disgust-sensitive individual has a 99% weekly chance of contact with pathogens. Under these circumstances, higher disgust sensitivity does little to decrease exposure to pathogens, and it imposes costs related to avoiding contact with conspecifics, who might be valuable sexual or social partners, avoiding potential foods, which might contain valuable nutrients and calories, and investing time and energy in avoiding physical locations. Rather than developing greater disgust sensitivity, then, individuals in higher parasite stress nations might navigate their pathogen-rich ecologies by investing more in tolerance or resistance (cf. [67]). Indeed, one study found that the Tsimane—an Amazonian foraging population living in the pathogen-rich lowlands of Bolivia—show higher anti-pathogen physiological signatures across an array of immunoglobulins, leucocytes and other inflammatory markers [68]. Of course, the consequences of contact with pathogens might be more severe in pathogen-rich ecologies, if the pathogens in such ecologies are more virulent, and the differences in contact probability between high and low disgust sensitivity individuals might have greater consequences in high versus low pathogen ecologies. And, further, cues might be more valid in high versus low pathogen ecologies (e.g. if faecal material is more likely to contain infectious microbes such as *Vibrio cholera* in such environments). Given these issues, predictions regarding the direction and strength of a relationship between ecological pathogen presence and disgust sensitivity are not straightforward.

The data described above speak against the hypothesis that national parasite stress relates to national aggregates of disgust sensitivity (and, hence, the possibility that cross-cultural relationships between parasite stress and variables such as religiosity, traditionalism and collectivism are mediated by disgust sensitivity [12]). However, they do not inform whether individual differences in disgust sensitivity within nations covary with individuals' capacity to resist infection (cf. [69]). Other research at the individual level can address this limitation.

### (b) Variability in disgust sensitivity as a function of individual vulnerability to pathogens

Psychologists have often relied upon retrospective accounts to measure infection frequency, recency and severity (e.g. [70]). One study of Australian students (*N* = 616) that used this approach found that disgust sensitivity was unrelated to both infection frequency and infection recency, and a measure of contamination sensitivity accounted for less than 4% of the variance in these same variables [71]. Of course, retrospective accounts are vulnerable to recall bias (e.g. disgust sensitivity influencing perceptions of infection frequency and recency) and random error.

Another study took a clever approach to circumventing this limitation. Using a sample of 284 Bangladeshi participants, De Barra *et al.* [72] assessed disgust sensitivity in adults and accessed those same adults’ childhood health records, which included monthly reports of diarrhoea and pneumonia up to age 5. The two variables were unrelated; individuals who suffered from more infectious disease in childhood had no higher disgust sensitivity in adulthood. The same participants reported their recent history of infection. As in the Australian student sample, retrospective accounts of recent infection histories were unrelated to disgust sensitivity. In total, then, the literature currently does not support the hypothesis that disgust sensitivity varies as a function of earlier infection. That said, this lack of a relationship is not necessarily inconsistent with the hypothesis that more infection-vulnerable individuals are more disgust sensitive. Indeed, such individuals' investments in avoidance might be successful, and hence they fall ill at a similar rate to those who are less vulnerable to infection [72].

Further, past infection is an imperfect predictor of current vulnerability. One notable line of work has tested whether another factor putatively related to infection vulnerability relates to disgust sensitivity: current progesterone. Based on women's downregulation of inflammatory immunity during the high progesterone luteal phase of the menstrual cycle, researchers have proposed that disgust sensitivity should be higher when progesterone levels are high [73,74]. One study of 79 naturally cycling female undergraduates found support for this hypothesis [74]. Progesterone, as measured via saliva, was positively correlated with self-reported disgust toward images portraying infectious disease risks and with self-reported contamination sensitivity over the past 24 h. However, a recent longitudinal study of 375 naturally cycling women found no relationship between changes in progesterone (also measured via saliva) and changes in disgust sensitivity over multiple assessments [75]. Another recent within-subjects study of 40 naturally cycling women found that disgust sensitivity was no higher in the luteal phase than in the follicular phase [76], and that changes in progesterone across the cycle were unrelated to changes in disgust sensitivity. That said, given the high stability of responses to disgust sensitivity instruments across time [75], such instruments might not be sensitive enough to capture intra-individual variation. Evidence for a relationship between progesterone and disgust sensitivity is thus equivocal.

In sum, our read of the literature suggests little-to-no relationship between disgust sensitivity and pathogens in the ecology, personal history of infectious disease or ability to resist pathogens. This conclusion contrasts with multiple hypotheses of variability in disgust [10,12,56]. Nevertheless, these hypotheses might be further tested with more precise methods (e.g. longitudinal and prospective) and measures (e.g. of immune markers). For example, work could test how disgust sensitivity relates to inflammatory responses or to time taken to recover from pathogen challenges (e.g. via lipopolysaccharide administration [77]).

## 5. Consequences of disgust sensitivity beyond pathogen avoidance

To this point, we have described data speaking against three prominent explanations for variability in disgust sensitivity—namely, that disgust sensitivity is a manifestation of broader neuroticism/emotionality, that it is shaped by parental modelling and that it is shaped by exposure to pathogens. So why do people vary in disgust sensitivity, then? Some perspectives suggest that random genetic mutation largely underlies variation in complex behavioural traits like disgust sensitivity rather than facultative calibration to an individual's condition [59] (cf. [57,58]). The lack of shared environmental effects in a twin study on disgust sensitivity [53] could speak against facultative calibration hypotheses, if the environmental factors shaping disgust sensitivity should be shared by twins (cleanliness of childhood residence), as could the small-to-zero relationship between ecological pathogen prevalence (and history of infection) and disgust sensitivity. Before embracing a random noise account of disgust sensitivity, though, researchers should generate and test alternative hypotheses. Such hypotheses can consider the costs and benefits of disgust sensitivity beyond those related to pathogen avoidance. We briefly overview three such consequences below.

### (a) Disgust sensitivity and nutritional stress

People avoid ingesting objects that elicit disgust [78] and learn to avoid foods that elicited nausea or disgust in the past [79,80], and the facial movements that co-occur with disgust expel ingested food and prevent other objects from entering the mouth [8]. Based on these (and other) observations, one prominent perspective argues that disgust evolved from earlier food choice adaptations [45] rather than from earlier pathogen-avoidance adaptations (cf. [2,5]). Regardless of the veracity of this proposal, the pathogen consequences of eating are clear: objects are more likely to cause infection if placed in the mouth than if left on the ground, on a plate, or in an offeror's hand. Indeed, to maximize pathogen avoidance, people should avoid putting anything in the mouth. Given that doing so would lead to starvation, the motivational structures underlying food choice should partially function to balance the pathogen and nutrition consequences of eating [81]. These consequences vary across individuals and contexts; as nutritional stress increases, the costs incurred by greater investment in avoiding pathogens also increase. Hence, we might see disgust sensitivity partially calibrated based on the current and expected future availability of calories.

A few studies have tested whether disgust toward cues to pathogens varies as a function of caloric state. One study randomly assigned participants to either fast for 15 h or eat a small lunch prior to a test session [16]. Those who had fasted made less intense disgust facial expressions toward typically unpalatable foods than those who were sated. Another study asked participants ‘How full (sated) do you feel right now?’ on a 7-point scale [82]. Less-sated participants scored lower on disgust sensitivity. And a further study reported that disgust sensitivity is negatively related to willingness to sample novel foods [83]. Of course, these studies have limitations (that reporting an effect of fasting on disgust toward unpalatable foods only included 44 participants in a between-subjects design; that reporting a relationship between satiation and disgust sensitivity found a standardized regression coefficient for the relationship between disgust sensitivity and hunger of *β* = 0.35, which is surprisingly large considering the high temporal stability of disgust sensitivity [75]), and hence should be replicated before forming the foundation of a theoretical framework for disgust sensitivity. Further, short-term food deprivation might not be enough to re-calibrate the stable variation observed in disgust sensitivity instruments. Future studies might examine whether responses to such instruments vary as a function of prolonged food scarcity or variation in phenotypic preparedness for food scarcity, which might be influenced by food stress during development or metabolic rate.

### (b) Disgust sensitivity and interdependence

Interdependence was an enduring feature of the human ancestral environment, and it has led to the evolution of multiple psychological adaptations dedicated to navigating social situations [84]. The benefits afforded by some forms of interdependence require physical contact between individuals—exactly the type of contact that poses pathogen risks to both members of a dyad (or all members of a larger group). Hence, some of the adaptations underlying interdependence likely monitor potential interaction partners for cues to infectiousness and use this information as a basis for partner choice and, in some cases, social exclusion [13]. As with caloric state, the benefits afforded by interdependence vary across individuals and situations. For example, in some situations, goal attainment requires two individuals to work in close proximity and coordinate actions; in other situations, one person's behaviour has no impact on another's outcomes and no coordination is required [84]. The former type of situation sometimes requires potentially infectious contact, whereas the latter rarely (if ever) does. Individuals might experience less disgust toward contact if they perceive themselves to be in a situation that requires contact for goal attainment (e.g. mutual dependence or correspondence, as opposed to independence or conflict). To test this idea, future studies could compare disgust toward close contact with others in interdependent, cooperative contexts (e.g. with team-mates during a team sport) with disgust in interdependent, competitive contexts (e.g. with an opponent during a tennis match) or independent contexts (e.g. between strangers sharing a sauna).

Reactions to contact with kin—and, specifically, offspring—provide a notable example of decreased disgust toward beneficial social contact. Anecdotally, parents experience little disgust toward physical contact with their offspring—contact that often transmits pathogens from infant to parent. Further, parents respond to contact with offspring vomit, faeces and urine with little revulsion. Indeed, one study reported that parents experience less disgust when smelling their own baby's soiled diaper than when smelling another baby's diaper, even when they were unaware of whose diaper they were smelling [85]. Similar examples can be observed within romantic dyads, where the type of intimate contact that would typically elicit disgust is instead embraced. These examples are target-specific, though—that is, experiencing less disgust toward contact with kin or mates might not correspond with less disgust toward cues to pathogens in the broader ecology. That said, some evidence does suggest that these same considerations might influence broader disgust sensitivity. For example, one paper comparing Slovak mothers (*N* = 174) with childless Slovak women (*N* = 124) found that mothers reported less disgust toward visual cues to pathogens and scored lower on the pathogen domain (but not the moral domain) of the Three Domain Disgust Scale [86]. This work suggests that the substantial increases in value of maternal–infant contact might have broader effects on disgust sensitivity.

Other work has investigated the relationship between disgust sensitivity and broader orientations toward cooperative contact. Two large-sampled studies (*N* = 477 and 476) found that agreeableness, which relates to the degree to which individuals perceive situations as characterized by corresponding rather than conflicting outcomes [87], relates negatively to disgust sensitivity, as measured by the Three Domain Disgust Scale [36,37] (though with weak effect sizes of *r* = −0.10 and −0.17, respectively). Further, pathogen disgust items related to social contact (e.g. shaking hands with a stranger who has sweaty palms) are more strongly related to agreeableness than are items related to non-social contact (e.g. stepping on dog poop) [88]. Higher pathogen disgust also relates to lower generalized social trust—that is, trust in people one does not know [89]—which in turn relates to cooperative behaviour, such as contributions to a public good [90]. That said, other perspectives have departed from that described here. Gangestad & Grebe [91], for instance, point out that individuals oriented toward frequent interactions with others (e.g. extraverted individuals) might benefit from high disgust sensitivity, because they have the highest probability of contact with socially transmitted pathogens, and thus should be most wary of cues to pathogens. They found evidence that more extraverted individuals indeed report greater disgust toward cues to human-transmitted pathogens, though other work has failed to replicate this finding [88]. Regardless of the existence of a relationship between extraversion and disgust sensitivity, Gangestad & Grebe's argument suggests another direction for future research: investigations into variability in pathogen cue detection rather than motivations to avoid pathogen cues when they are detected (i.e. disgust sensitivity). Individuals with social orientations that involve more physical contact might invest more attentional resources in detecting pathogens rather than motivations to avoid them.

### (c) Disgust sensitivity and sexual strategy

Sexual interactions pose the highest infectious disease risk of any social behaviour. They involve physical contact of the skin, the genitals, and often the mouth and hair, and they subsequently expose individuals to multiple types of bodily fluids (e.g. saliva, semen, vaginal fluids, blood), each of which can transmit pathogens [2]. As with eating, though, maximal pathogen avoidance (i.e. never having sex) would have debilitating fitness consequences, and people accept some pathogen risk to facilitate reproduction. Consequently, the disgust typically experienced at the thought of a stranger's tongue in one's mouth melts away if that stranger is replaced by one's romantic partner (or, for some people, by a particularly attractive stranger). This latter point is important. Whereas few people would recoil from the thought of close, potentially infectious contact with their pair-bonded partner, individuals vary markedly in their openness to sex outside of a pair-bonded relationship—that is, they vary in sociosexuality [92]. Such variability relates to disgust sensitivity, with more pathogen-avoidant individuals being more sociosexually restricted (i.e. less open to sex outside of a relationship) [93].

The relationship between disgust sensitivity and sociosexuality has been interpreted as suggesting that individuals pursuing a more monogamous orientation do so partially to avoid the increasing pathogen costs that accompany each new sexual partner [93,94]. This interpretation is consistent with work showing that sociosexuality is lower in nations with more infectious disease [60] and among individuals with higher disgust sensitivity. However, causation could flow in the opposite direction. Given that a successful unrestricted sociosexual strategy requires intimate contact with multiple individuals, unrestricted individuals might adopt lower disgust sensitivity to facilitate their mating strategies. Further, as with personality, sociosexuality might also covary with vigilance toward pathogen cues in addition to—or rather than—motivations to avoid such cues. As with many of the hypotheses discussed here, future work is necessary to disentangle these possibilities.

## 6. Summary

Psychologists have conducted an impressive amount of research on disgust sensitivity over the past 20 years. The time is ripe to use these data to critically evaluate assumptions of how to interpret this variability. Here, we identified three interpretations of this variation: (i) that disgust sensitivity is a component of neuroticism/emotionality; (ii) that it emerges via parental modelling; and (iii) that it is calibrated to the presence of infectious disease in the local ecology and/or personal vulnerability to infection. Based on our review of the literature, little evidence supports these proposals, and much evidence contradicts them. Our interpretations of the data (if correct) suggest that new endeavours should be taken to better understand the roots of disgust sensitivity. We have also laid out a few hypotheses here—namely, that disgust sensitivity might emerge based on people's sexual, nutritional and social requirements or strategies. Myriad additional hypotheses surely remain to be articulated and tested. We hope that this summary has provided some inspiration for generating and testing such hypotheses and, ultimately, understanding this critical aspect of human pathogen avoidance.

## Endnotes

1 Note that, for the purpose of this paper, we use the term ‘disgust sensitivity’ to refer to pathogen disgust. Other types of disgust sensitivity—specifically, sexual and moral disgust—likely emerge for different reasons, which would require a separate paper to address (though see [17] and [18] for some discussion of these issues).

2 Statistical power was estimated using G*Power 3.1.9.2., with a test between two independent correlations, an effect size of *q* = 0.254 (*ρ*_1_ = 0.41, *ρ*_2_ = 0.18), and sample sizes of 38 and 34. Based on the effect size reported by Sherlock *et al.* [53], power to detect genetic effects in Rozin & Millman's study [49] was 0.23.

## Data accessibility

This article has no additional data.
